# A Novel Clinical Decision Support System Using Improved Adaptive Genetic Algorithm for the Assessment of Fetal Well-Being

**DOI:** 10.1155/2015/283532

**Published:** 2015-02-22

**Authors:** Sindhu Ravindran, Asral Bahari Jambek, Hariharan Muthusamy, Siew-Chin Neoh

**Affiliations:** ^1^School of Microelectronic Engineering, Universiti Malaysia Perlis (UniMAP), Campus Pauh Putra, 02600 Perlis, Malaysia; ^2^School of Mechatronic Engineering, Universiti Malaysia Perlis (UniMAP), Campus Pauh Putra, 02600 Perlis, Malaysia; ^3^Department of Computing Science and Digital Technologies, University of Northumbria, Newcastle NE, UK

## Abstract

A novel clinical decision support system is proposed in this paper for evaluating the fetal well-being from the cardiotocogram (CTG) dataset through an Improved Adaptive Genetic Algorithm (IAGA) and Extreme Learning Machine (ELM). IAGA employs a new scaling technique (called sigma scaling) to avoid premature convergence and applies adaptive crossover and mutation techniques with masking concepts to enhance population diversity. Also, this search algorithm utilizes three different fitness functions (two single objective fitness functions and multi-objective fitness function) to assess its performance. The classification results unfold that promising classification accuracy of 94% is obtained with an optimal feature subset using IAGA. Also, the classification results are compared with those of other Feature Reduction techniques to substantiate its exhaustive search towards the global optimum. Besides, five other benchmark datasets are used to gauge the strength of the proposed IAGA algorithm.

## 1. Introduction

In clinical practice, cardiotocography (CTG) was introduced in the late 1960s, which is a noninvasive and cost-effective technique for evaluating the fetal well-being. This technique has been widely used by obstetricians for examining the fetal well-being (inside the mother's uterus) as the fetus is not available for direct observations. The baby's fetal heart rate (FHR) and the mother's uterine contractions (UC) are recorded on a paper trace known as cardiotocograph. The CTG technique is highly instrumental in the early identification of a pathological state (i.e., congenital heart defect, fetal distress, or hypoxia) and it helps the obstetrician to predict future complications.

During the critical period of labor, these FHR signals are used as a denotation of the fetal condition and as a warning of possible fetal and neonatal compromise, namely, metabolic acidosis. Severe hypoxic injury of the fetus can result in the neurodevelopmental disability and cerebral palsy or even death [1]. Hence, such FHR patterns are devised in such a way that such risky conditions of the fetus need to be identified in earlier stages, in order to alert the obstetricians to intervene before there is an irreversible damage to the fetus. Although the FHR signals interpret and provide early estimations and warnings about the fetal condition [2], still, there has been lot of scepticism as there is inconsistency in such interpretation and the increase of false positive diagnosis. On one hand, advances in signal processing and pattern recognition techniques have paved the way to develop an efficient medical diagnosis system to analyze and classify the FHR signal appropriately [3].

In this paper, a clinical decision support system called Improved Adaptive Genetic Algorithm (IAGA) has been developed to discern the FHR signals of the CTG recordings into their respective groups. For this purpose, the CTG dataset has been acquired from the UCI machine learning repository for experimentation. This dataset consists of 2126 CTG samples and each of these samples has a feature length of 21. Out of these 2126 samples, 1655 samples belong to the normal state, 176 samples belong to the pathological state, and 295 samples belong to the suspect state. Features of this CTG dataset used for experimentation have been explained in Table 1. Single and multiobjective fitness functions have been used to gauge the efficacy of the IAGA. Several classifiers like *k*-NN (*k*-Nearest Neighbor), SVM (Support Vector Machine), BN (Bayesian Network), and ELM (Extreme Learning Machine) have been used to discern these features.

With the objective of proving the robustness of this search algorithm, apart from the CTG dataset, five other benchmark datasets, that is, MEEI voice dataset, Parkinson's (PD) dataset, Cleveland Heart Disease (CAD) dataset, Erythemato-Squamous (ES) dataset, and Breast Tissue (BT) dataset, have been taken from the UCI machine learning repository for experimentation and tested with this algorithm. The main aim is to select the best clinical features of these datasets through the proposed IAGA method, which, in turn, attains promising classification accuracy with a minimal number of features.

The rest of this paper is organised as follows.  Section 2 describes the literature works done in this specific problem domain.  Section 3 explains about the feature selection carried out using IAGA.  Section 4 presents the classifiers employed and the performance measures.  Section 5 enunciates the discussion of the classification results of CTG dataset.  Section 6 elaborates the classification results of other datasets and Section 7 concludes the entire work.

## 2. Previous Works on CTG Dataset

Numerous approaches have been investigated using conventional and artificial intelligence techniques for feature extraction and also to come out with diagnostic systems [4]. In [3], the automatic classification of FHR signal which belongs to hypoxic and normal newborns has been carried out through a hidden Markov models (HMM) based approach. Yet again, an ANBLIR (artificial neural network based on logical interpretation of fuzzy if-then rules) system is used to evaluate the risk of low-fetal birth weight as normal or abnormal using CTG signals recorded during the pregnancy in [5].

### 2.1. Basic GA

As an expeditious search strategy, GA has been utilized in the assessment of the fetal well-being. For example, Ocak (2013) reported that a genetic based optimization followed by SVM classification helps in predicting the substantial features for assessing the fetal state. The classification accuracy with FHR data set obtained was 99.23% with 13 features [6]. An adaptive neurofuzzy inference system (ANFIS) has been proposed for the prediction of fetal status from the CTG recordings as normal or pathological [7].

Yılmaz and Kılıkçıer have suggested a combined scheme of binary decision tree (BDT) and particle swarm optimization (PSO) for handling this specific classification task. Through least squares support vector machine (LS-SVM), a good classification accuracy rate of 91.62% was achieved. Besides, this experimentation has resulted in the three-class classification of the CTG dataset using receiver operation characteristic analysis and Cobweb representation [8]. However, the problem of design of a better genetic based search heuristic that provides higher classification accuracy along with a reduced number of voice features in this specific problem domain is still open. In other words, the reduced optimal feature subset must be sufficient enough to classify the data samples into their respective classes than the previous works.

**Figure 1:**
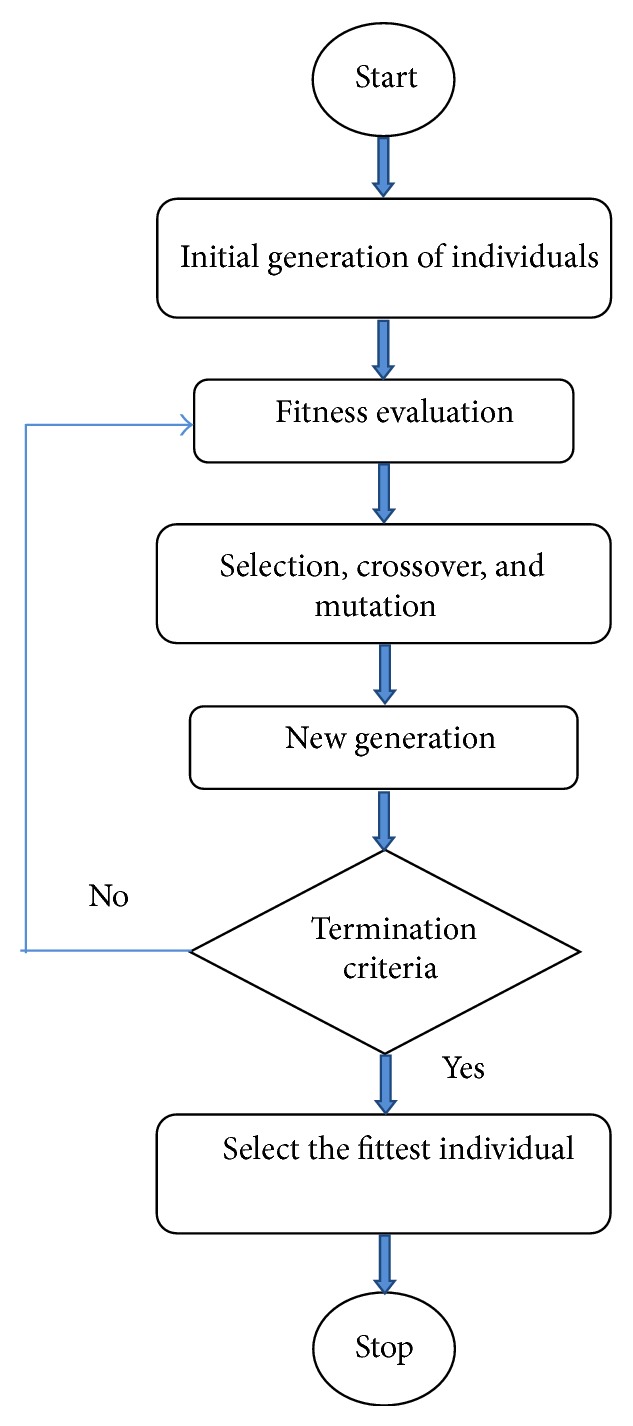
Basic GA cycle.

Basic GA deals with candidate solutions which are represented by individuals (or chromosomes) in a large population. It starts with the random generation of initial set of chromosomes followed by their corresponding fitness evaluation. The successive generations are created (iteratively) by the picking of the highly fit individuals of the current generation. On achieving the eligible individual that satisfies our constraint, the GA cycle is stopped using the termination criteria and this individual becomes the solution of the problem. There are three significant genetic operators called selection, crossover, and mutation, which help in the reproduction process of the chromosomes [9]. Ideally, a GA cycle takes place using these genetic operators and such genetic parameters influencing this process tend to produce good individuals. Such a basic GA cycle is depicted in Figure 1.

## 3. An Improved Adaptive Genetic Algorithm (IAGA) for Feature Selection

To begin with, initially, the input data of the CTG dataset is extracted from the FHR signals. IAGA selects the best optimal feature subset which is then fed as inputs to the classifiers and these classifier inputs are distinguished into their respective number of classes. The classification results are presented in terms of performance measures like Positive Prediction, Negative Prediction, Sensitivity, Specificity, Area Under Receiver Operating Curve (AUC), Overall Accuracy, *F*-measure, *G*-mean, and Kappa statistic, and so forth. Besides, the performance of the IAGA method has been compared with three existing feature reduction and feature selection methods (PCA, SFS, and SBS) so as to affirm the strength of proposed search algorithm.

Despite the algorithm of Basic GA being applied to this CTG dataset, IAGA is implemented in two different ways with certain modification in the crossover and mutation probabilities and their mode of usage during the search procedure. Totally, three types of FS (Basic GA, IAGA-method 1, and IAGA-method 2) have been performed on this dataset followed by feature classification. Initially, Basic GA is employed to this CTG dataset for optimizing the features, which was mentioned earlier in Section 2. This is further improved as IAGA for the same purpose, which varies from Basic GA in terms of selection function, crossover and mutation rates, and fitness functions. Three different fitness functions have been formulated as the evaluation measures of IAGA.

### 3.1. Stochastic Universal Sampling

The proposed IAGA method employs the stochastic universal sampling (SUS) technique for selecting the fittest chromosomes. This technique was developed by Baker in 1987 [10]. Based on this random search, the SUS function utilizes a single random value to sample the chromosomes. It makes use of several strategies of GA to select the best parent chromosomes for reproduction. Also, it ensures that these chromosomes are highly capable of being reproduced. Through this selection scheme, the genetic diversity of the population is highly maintained.

### 3.2. Sigma Scaling

An important parameter that must be fine-tuned during the genetic search is the selection pressure (SP), that is, the degree to which the selection emphasizes the better chromosomes. When this SP is very low, it will result in the low convergence rate of the search algorithm. On the other hand, a higher SP will make the search procedure to attain premature convergence easily. Hence, to overcome such conditions, various fitness scaling methods (a method used to adjust the chromosomal fitness) have been proposed to prevent GA from attaining the premature convergence. Sigma scaling is one such a method that maintains a constant SP throughout the entire generation and it is used in this IAGA method for the reorientation of chromosomal fitness. Suppose *f*_*x*_^(*t*)^ is the fitness of some individual *x* of generation *t* and suppose the average fitness and standard deviation (SD) of the fitness of the individuals in generation *t* are given by *f* ^(*t*)^ and *σ* ^(*t*)^, respectively; then the adjusted fitness of *x* in generation *t* is given as follows:

(1) $$\begin{array}{c} h_{x}^{\left(t\right)}=\left\{\begin{array}{cc} \min\left(0,1+\frac{f_{x}^{\left(t\right)}-\bar{f^{\left(t\right)}}}{\sigma^{\left(t\right)}}\right), & \sigma^{\left(t\right)}\neq 0\\ 1, & \sigma^{\left(t\right)}=0. \end{array}\right. \end{array}$$

### 3.3. Adaptive Crossover and Mutation with Masking

Uniform crossover is applied in the search procedure of IAGA with a crossover rate (*P*_*c*_) of 0.9. During uniform crossover, each gene in the offspring is created by copying the corresponding gene from one or the other parent, chosen according to a randomly generated crossover mask. A single crossover mask is randomly generated during the search of IAGA for each pair of parents (P1—first parent and P2—second parent), in which every “1” and “0” in the mask imply the copied gene from the first parent and second parent, respectively [11]. The offspring produced in this way constitutes a collection of genes from both the parents.

Flip-bit mutation is applied with a mutation rate (*P*_*m*_) of 0.03. The mutation operator makes use of a random mask. It changes those corresponding bits which imply “1” in the mask. Depending upon this random nature of the mask, the value of *P*_*m*_ is determined and this highly influences the random behavior of the *P*_*m*_ value [12]. However, it is good to vary the mutation rate between 0.1 and 0.001.

### 3.4. Fitness Functions

#### 3.4.1. Objective Function I

Three fitness functions have been devised to assess the chromosomal quality and they are given below

(2) $$\begin{array}{c} \text{Objv}1=\text{Accuracy}. \end{array}$$

#### 3.4.2. Objective Function II

During the classification of binary and multiclass problems, error rate and classification accuracy are the two popular measures used for evaluating the classifier performance. Generally, the classifier's accuracy is determined as its performance measure in balanced datasets [13]. But when dealing with imbalanced data, accuracy is known to be unsuitable to measure the classification performance, as it may mislead the classification process due to the emphasis on the influence of the majority class.

In order to overcome this convenience, a very few metrics have been devised as fitness functions and they are geometric mean (*G*-mean), AUC (area under ROC curve), and *F*-measure. In this approach of IGA, geometric mean (*G*-mean) has been chosen as an objective function as it is one of the most popular measures for handling imbalanced data and easier to calculate than *F*-measure and AUC. In this regard, a new fitness function (Objv2) has been devised to evaluate the fitness, where the classification performance will be evaluated through geometric mean and its equation is defined as

(3) $$\begin{array}{c} \text{Objv}2=\overset{c}{\underset{i=1}{\prod }}{\left(M_{i}\right)}^{1/c}, \end{array}$$

where

(4) $$\begin{array}{c} M_{i}=\frac{\text{Number}\;\text{of}\;\text{correctly}\;\text{classified}\;\text{samples}}{\text{Total}\;\text{number}\;\text{of}\;\text{samples}}\\ c=\text{number}\;\text{of}\;\text{classes}\;\text{in}\;\text{the}\;\text{dataset}. \end{array}$$

For binary class, the value of *c* = 2 and it corresponds to classify the data into two groups. For multiclass, the value of *c* = 1,2, 3,…, *N* and it corresponds to classify the data into *N* number of classes.

#### 3.4.3. Objective Function III

Eventually, in this IAGA approach, a single objective fitness function (Objv2) has been combined with a multiobjective fitness function (number of zeros in the chromosome) to bias the genetic search towards the global optimum and it is defined as

(5) $$\begin{array}{c} \text{Objv}3=\left(w\right)\text{Objv}2+\left(1-w\right)\left(\frac{Z}{N}\right), \end{array}$$

where Objv2 corresponds to the *G*-mean value and *w*  (0 < *w* < 1) is the equalizing factor, which adjusts the significance of *G*-mean, *Z* implies the number of zeros in the chromosome, and *N* implies the length of the chromosome.

### 3.5. Methods of IAGA

#### 3.5.1. IAGA-Method 1

The adaptive approach of IAGA has been devised in two different ways. In basic GA, when the values of *P*_*c*_ and *P*_*m*_ are maintained constantly throughout the entire search procedure, there will be no improvement in the individuals' fitness or it may result in premature convergence, owing to attain a suboptimal solution. This not only will affect the performance of search algorithm but also fails to achieve the desired solution expeditiously. This can be avoided by modifying the values of *P*_*c*_ and *P*_*m*_ in an adaptive manner in accordance with the chromosomal fitness in their specific generations [14].

Based on the convergence to the optimum, this IAGA-method 1 (IAGA-M1) determines the adaptive crossover and mutation rates. This is done with the help of certain measures like average fitness value (*f*_avg_) and maximum fitness value (*f*_max⁡_) of the population, respectively. *X* gives this relationship between the maximum and average fitness as follows:

(6) $$\begin{array}{c} X=f_{\max}-f_{\text{avg}}. \end{array}$$

However, when GA converges to local optimum, that is, when the value of *X* decreases, the values of *P*_*c*_ and *P*_*m*_ have to be increased. Inversely, when the value of *X* increases, these values have to be decreased. Besides, when GA converges to a locally optimal or even globally optimal solution, the increase being done in the values of *P*_*c*_ and *P*_*m*_ will disrupt the near-optimal solutions [14]. Due to this effect, the population may never converge to the global optimum and the performance of GA will be diminished considerably.

In order to overcome these issues, two more fitness measures called *f*′ and *f*′′ have been taken into account in such a way that these measures help to preserve the excellent individuals of the population. *f*′ is the bigger fitness of the two crossover chromosomes and *f*′′ is the fitness value of the individual that has to be mutated. These measures are highly instrumental in overcoming the premature convergence and preserving the excellent individuals. Previously, the values of *P*_*c*_ and *P*_*m*_ are varied depending upon the *X* value. But now, we can conclude that *P*_*c*_ and *P*_*m*_ are not only related to *X* but also related to *f*_max⁡_ − *f*′ and *f*_max⁡_ − *f*′′ [15]. Eventually, the *P*_*c*_ and *P*_*m*_ values are determined using the following equation as

(7) $$\begin{array}{c} P_{c}=\left\{\begin{array}{cc} \frac{k_{1}\left(f_{\max}-f^{\prime }\right)}{\;\left(f_{\max}-f_{\text{avg}}\right)} & f^{\prime }\geq f_{\text{avg}}\\ 0.9 & f^{\prime }<f_{\text{avg}} \end{array}\right.\\ P_{m}=\left\{\begin{array}{cc} \frac{k_{2}\left(f_{\max}-f^{\prime \prime }\right)}{\;\left(f_{\max}-f_{\text{avg}}\right)} & f^{\prime \prime }\geq f_{\text{avg}}\\ 0.03 & f^{\prime \prime }<f_{\text{avg}}. \end{array}\right. \end{array}$$

For both these equations in (7), *k*_1_ and *k*_2_ are predetermined values that are less than 1.0 considering the probability for mutation and crossover [14]. In this experiment, the values of *k*_1_ and *k*_2_ have been empirically determined and fixed as 0.4 and 0.1, respectively.

#### 3.5.2. IAGA-Method 2

In IAGA-method 2 (IAGA-M2), the values of *P*_*c*_ and *P*_*m*_ are devised with their maximum and minimum probabilities; that is, instead of applying two values for *P*_*c*_ and *P*_*m*_, four values, namely, *P*_*c*max⁡_, *P*_*c*min⁡_, *P*_*m*max⁡_, and *P*_*m*min⁡_ are infused. This adaptive approach has been followed in order to maintain diversity, thereby sustaining the convergence capacity of IAGA [16]. Hence, the crossover and mutation rates are chosen as described in the following equation:

(8) Pc=Pcmax⁡−Pcmax⁡−Pcmin⁡1+exp⁡λf′−favg/fmax⁡−favgf′≥favgPcmax⁡f′<favgPm=Pmmax⁡−Pmmax⁡−Pmmin⁡1+exp⁡λf′−favg/fmax⁡−favgf′≥favgPmmax⁡f′<favg,

where *f*_max⁡_ is the maximum fitness, *f*_avg_ is the average fitness of the population, *f*′ is the bigger fitness of the two crossover chromosomes, and *f*′′ is the fitness value of the individual that has to be mutated; *P*_*c*max⁡_ and *P*_*c*min⁡_ are the maximum and minimum probabilities of crossover, *P*_*m*max⁡_ and *P*_*m*min⁡_ are the maximum and minimum probabilities of mutation [16], and lambda is a constant (*λ* = 2).

**Figure 2:**
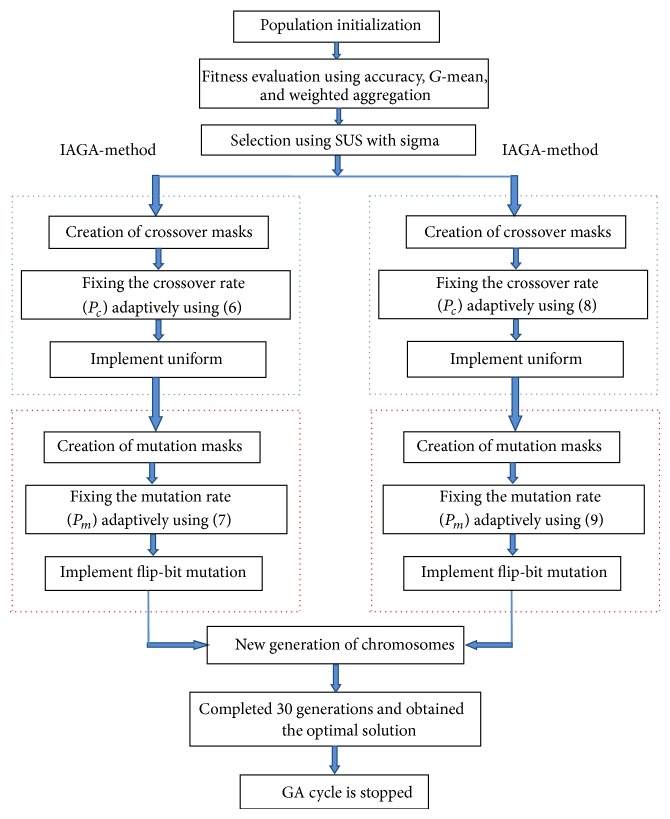
Genetic cycle using IAGA-methods 1 and 2.

While modifying the crossover and mutation rates, the values of *P*_*c*max⁡_ and *P*_*c*min⁡_ are chosen as 0.9 and 0.6 on the basis of empirical methods. Similarly, the values of *P*_*m*max⁡_ and *P*_*m*min⁡_ are also chosen as 0.1 and 0.001 based on empirical methods, respectively. Hence, when the chromosomes are subjected to crossover and mutation, the values of *P*_*c*_ and *P*_*m*_ are modified adaptively and then masking is applied. The specifications of IAGA are tabulated in Table 2 and Figure 2 shows the entire genetic cycle using the two methods of IAGA.

## 4. Experimental Setup

### 4.1. Classifiers

The performance of the classifiers employed in this work is examined by 10-fold cross validation scheme, which have served as an evaluator of this proposed IAGA algorithm. For the classification of selected features, four linear and nonlinear classifiers like *k*-NN, SVM, BN, and ELM are applied. For performing binary classification, both *k-*NN and SVM are used, in which SVM gives better classification results. For multiclass classification, *k*-NN, BN, and ELM are employed, wherein ELM achieves better classification performance. A detailed description of these classifiers is discussed below.

#### 4.1.1. Support Vector Machine

Support vector, machine (SVM) is an acknowledged classification technique, being widely used for solving classification problems. In general, SVM separates the classes with a decision surface that increases the margin between the classes. The surface is called the optimal hyperplane and the data points closest to this hyper plane are called support vectors [17]. The data used in this work is not linearly separable. Hence, nonlinear kernel functions are employed to transform the data into a new feature space where a hyperplane tends to separate the data. The optimal separating hyperplane is being searched by these kernel functions in a new feature space to increase its distance from the closest training point. Due to a better generalization capability and low computational cost, RBF kernel has been applied in this work for separating the optimal hyperplane and the equation of the kernel function is given below

(9) $$\begin{array}{c} K\left(x,x_{i}\right)=\exp\left(\frac{-{\left\|x-x_{i}\right\|}^{2}}{2\sigma^{2}}\right). \end{array}$$

There are two significant parameters called regularization parameter (*γ*) and squared bandwidth of the RBF kernel (*σ* ^2^), which should be optimally selected to obtain promising classification accuracy. By trial and error, the value of sigma (*σ* ^2^) and gamma (*γ*) for the RBF kernel was set to 0.4 and 90, respectively.

#### 4.1.2. Extreme Learning Machine

A new learning algorithm for the single hidden layer feed forward networks (SLFNs) [18] called extreme learning machine (ELM) was proposed by Huang et al. It has been widely used in various applications to overcome the slow training speed and overfitting problems of the conventional neural network learning algorithms [19]. For the given *N* training samples, the output of a SLFN network with *L* hidden nodes can be expressed as the following:

(10) $$\begin{array}{c} f_{L}\left(x_{j}\right)=\overset{L}{\underset{i}{\sum }}\beta_{i}g\left(w_{i}\cdot x_{j}+b_{i}\right),\;j=1,2,3,\ldots ,N. \end{array}$$

It can be described as *f*(*x*) = *h*(*x*)*β*, where *x*_*j*_, *w*_*i*_, and *b*_*i*_ are the input training vector, input weights, and biases to the hidden layer, respectively. *β*_*i*_ is the output weights that links the *i*th hidden node to the output layer and *g*(·) is the activation function of the hidden nodes.

Training an SLFN is simply finding a least square solution by using Moore-Penrose generalized inverse:

(11) $$\begin{array}{c} \hat{\beta }=H†T, \end{array}$$

where *H*† = (*H*′*H*)^−1^ *H*′ or *H*′(*HH*′)^−1^, depending on the singularity of *H*′*H* or *HH*′. Assume that *H*′*H* is not a singular and the coefficient 1/*ε* (*ε* is positive regularization coefficient) is added to the diagonal of *H*′*H* in the calculation of the output weights *β*_*i*_. Hence, more stable learning system with better generalization performance can be obtained. The output function of ELM can be written compactly as

(12) $$\begin{array}{c} f\left(x\right)=h\left(x\right)H^{\prime }{\left(\frac{1}{\varepsilon }+HH^{\prime }\right)}^{-1}T. \end{array}$$

During the implementation of ELM kernel, the hidden layer feature mappings need not to be known to users and RBF kernel has been employed in this work. Best values for positive regularization coefficient (*ε*) as 4 and RBF kernel parameter as 1 were found empirically after several experiments.

### 4.2. Implementation of the IAGA with Benchmark Datasets

The implementation of the proposed IAGA methods along with the respective FS and FR methods has been elaborated in this section. The entire process takes place in three major steps which are

- feature reduction/selection using four existing methods,
- optimization using IAGA method,
- classification through *k*-NN, SVM, BN, and ELM.

Initially, PCA is applied to the data samples of the concerned dataset and their dimensionality is reduced to the desired range. From PCA, only the principal components of having 98% of the variability are selected as features. Simultaneously, SFS and SBS are employed with the same dataset to select the minimized feature subset. In SBS and SFS, only 50% of the features are selected from optimally ordered set of all features of the six datasets. The reduced feature subsets of both these methods are individually fed as inputs to the classifiers.

Secondly, the proposed IAGA method is applied for choosing the best features of the specific dataset. This method involves certain significant levels of the genetic process like population initialization, chromosome selection using SUS with sigma scaling, applying crossover and mutation using masking techniques, fitness evaluation using the three fitness functions (Objv1, Objv2, and Objv3), and satisfying the termination criteria. The selected feature subset obtained through this optimization technique is fed as inputs to the classifiers. Hence, three types of inputs are sent to the classifiers (inputs from PCA, inputs from SFS and SBS, and reduced inputs from IAGA).

**Figure 3:**
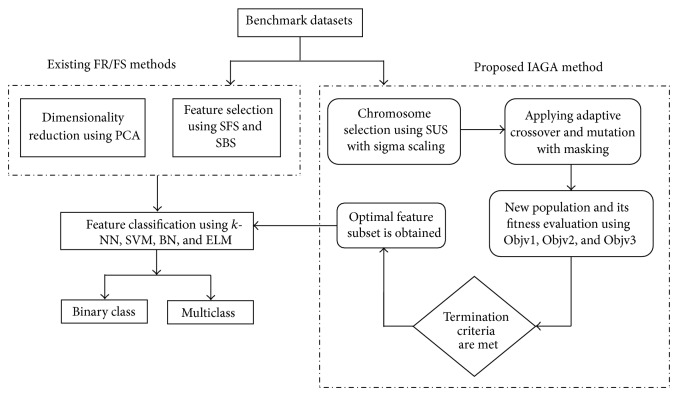
Implementation of IAGA in pattern classification.

Finally, the classifier tends to classify the inputs into their respective binary or multiclass groups based on the number of the classes of the concerned dataset. For instance, when CTG dataset is subjected to the aforementioned processes, classifiers like *k*-NN, BN, and ELM will classify the data samples into pathological, suspect, and normal samples. Similarly, when PD dataset is subjected to the aforementioned processes, the *k*-NN and SVM classifiers will classify the data samples into pathological and normal samples.  Figure 3 describes the implementation of the proposed IAGA methods along with the benchmark datasets.

### 4.3. Performance Measures-Confusion Matrix

A confusion matrix is a plot used to evaluate the performance of a classifier. As the proposed IAGA algorithm deals with both binary and multiclass classification problems, the experimental results are presented in terms of several performance measures. Among these measures, the classification results of binary classification are explained in terms of Sensitivity, Specificity, AUC, Overall Accuracy, *F*-measure, *G*-mean, and Kappa statistic. The experimental results of multiclass classification are described in terms of Accuracy and *G*-mean. True positive (TP), true negative (TN), false positive (FP), and false negative (FN) are the four basic elements of a confusion matrix and the aforementioned performance measures are evaluated from these elements, which are explained below.

 Sensitivity (SE):
  (13) $$\begin{array}{c} \text{SE}=100\times \frac{\text{TP}}{\text{TP}+\text{FN}}. \end{array}$$
 Specificity (SP):
  (14) $$\begin{array}{c} \text{SP}=100\times \frac{\text{TN}}{\text{TN}+\text{FP}}. \end{array}$$
 Accuracy (ACC):
  (15) $$\begin{array}{c} \text{ACC}=100\times \frac{\text{TP}+\text{TN}}{\text{TP}+\text{TN}+\text{FP}+\text{FN}}. \end{array}$$
 
*F*-measure:
  (16) $$\begin{array}{c} F\text{-measure}=\frac{2\times \text{precision}\times \text{recall}}{\text{precision}+\text{recall}}, \end{array}$$
 where
  (17) $$\begin{array}{c} \text{precision}=\frac{\text{TP}}{\text{TP}+\text{FP}},\;\;\text{recall}=\frac{\text{TP}}{\text{TP}+\text{FN}}. \end{array}$$
 Area under ROC:
  (18) $$\begin{array}{c} \text{ROC}=\frac{\text{SE}+\text{SP}}{2}. \end{array}$$
 Kappa statistic:
  (19) $$\begin{array}{c} \text{KS}=\frac{P_{0}-P_{c}}{1-P_{c}}, \end{array}$$
 where *P*_0_ is the total agreement probability and *P*_*c*_ is the hypothetical probability of chance agreement.

 
*G*-mean:
  (20) $$\begin{array}{c} G\text{-mean}=\overset{c}{\underset{i=1}{\prod }}{\left(M_{i}\right)}^{1/c}. \end{array}$$

## 5. Results and Discussion

### 5.1. Classification Results of CTG Dataset

Pattern classification is performed on the CTG data by employing IAGA methods to perform FS. In addition, this dataset is also treated with the three existing FS methods like PCA, SFS, and SBS and their simulation results are compared with those of the IAGA methods.  Table 3 presents the comparison of simulation results obtained after applying the proposed IAGA methods and three existing FS methods. It can be seen from this table that, from PCA, 16 principal components containing 98% of the variability are selected.

When FS is performed using SBS and SFS, first 11 features (50% of the features) are selected from the optimally ordered set of 22 features. Totally, the simulation results unfold that the three existing FS methods have brought out a best average classification accuracy of 92.14%, 92.10%, and 92.71%, respectively. In the view of comparing the classification performance of the IAGA methods directly with that of the three existing FS methods (PCA, SBS, and SFS), first 6 features are chosen and classification experiments are conducted. The classification accuracy obtained by selecting the first 6 features using PCA, SBS, and SFS is 89.84%, 91.44%, and 90.64%, respectively.

**Figure 4:**
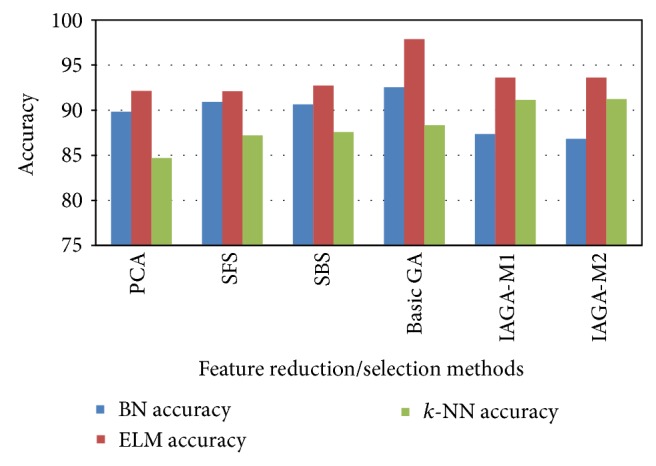
Classification accuracies of all the FR/FS methods using CTG dataset.

**Figure 5:**
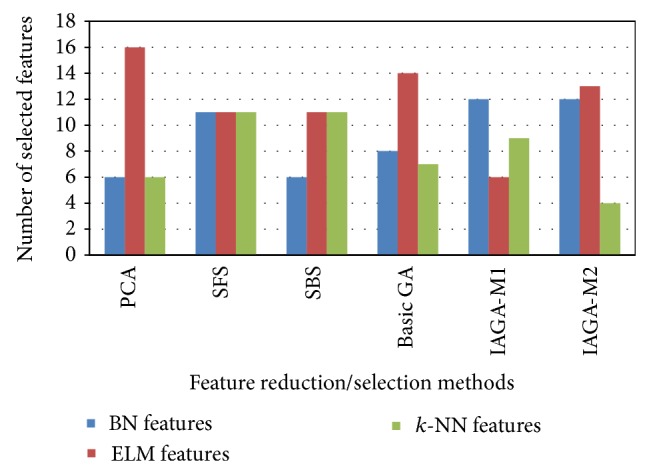
Number of selected features using all the FR/FS methods for CTG dataset.

Figures 4 and 5 show the comparative view of the classification accuracies and number of selection features using all the FR/FS methods for the CTG dataset. The inference from these Figures reveals that, among all the FR/FS methods, the overall (best) classification accuracy is yielded through IAGA-M1 for 6 features.

Table 4 explains the performance measures of all the FR/FS methods using this dataset. The observations show that the two IAGA methods have come out with a moderate accuracy ranging between 85% and 95%. However, when the overall performance is taken into account, the best average classification accuracy has been achieved by both IAGA-M1 and M2 with 93.61%. But when comparing the feature count, IAGA-M1 has accomplished better results than IAGA-M2 by selecting only 6 CTG features and those six features are LB (FHR baseline), AC (accelerations), UC (uterine contractions), ASTV (abnormal short term variability), ALTV (abnormal long term variability), and MLTV (mean value of long term variability). Also, these two methods have produced best *G*-mean values of 85.57% and 85.88% through ELM classification.

Table 5 represents the classification results obtained before and after undergoing FS procedure. At the initial stage of classification, when the original CTG samples are directly fed into the ELM classifier, the accuracy range obtained was mediocre. However, when these features are selected through IAGA, the accuracy seemed to increase by 2.64% significantly. Eventually, the maximum classification accuracy of 94.01% is obtained using IAGA-M1 for the discrimination of the CTG samples with the SD of 0.24.

### 5.2. Confusion Matrix of CTG Dataset

Table 6 explicates the simulation results of CTG dataset in the form of confusion matrix for the three classifiers. Since 3 class classifications have been performed on this dataset, there seems to be moderate amount of misclassifications for all the three groups. Out of the 1655 pathological samples, 1620 samples were correctly classified with remaining 35 incorrect classifications. For the 295 suspect samples, there are 224 correct classifications with 71 misclassifications. Finally, out of 176 normal samples, 152 normal samples are correctly classified with the remaining 24 incorrectly classified samples.

## 6. Classification Results of Other Benchmark Datasets

### 6.1. Comparison with Previous Works for All the Datasets

The observation from Table 7 shows the comparison of the classification results of CTG dataset and the other five datasets with their previous works in the literature. Inferences from the literature enunciate that the proposed IAGA technique has achieved the best classification accuracies (e.g., 93.61% for CTG dataset) and optimal feature subset (6 clinical features) so far. The classification performance has been achieved similarly for the remaining datasets as well.

### 6.2. Overall Classification Performance

**Figure 6:**
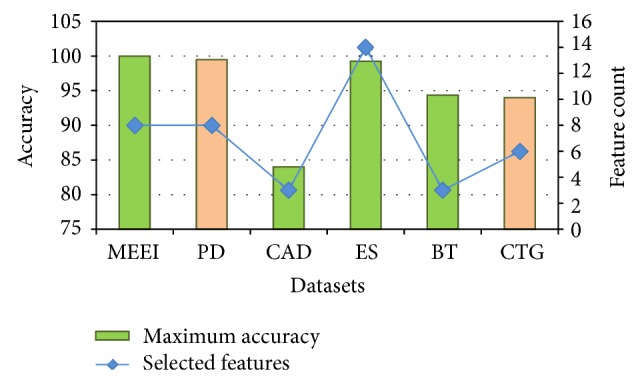
Comparison plot of overall performance of six datasets.

The overall classification performance is displayed in the form of comparison plot in Figure 6 and this figure substantiates the maximum classification performances achieved using the proposed search techniques. When analysing the overall performance, the best classification accuracy has been achieved through IAGA-M2 for four datasets and IAGA-M1 for two datasets.

### 6.3. Comparison of Fitness Functions of the IAGA Methods

Three fitness functions, namely, classification accuracy, *G*-mean, and weighted aggregation (*G*-mean + sum of unselected features), have been devised as the first, second, and third fitness function, respectively.  Table 8 depicts the overall comparison between these three different fitness functions of the proposed IAGA methods. The overall observation from this table implies that the third fitness function (Objv3) (indicated by the symbol ***√***) of all the three proposed methods has produced the maximum number of best classification accuracies for most of the datasets.

## 7. Conclusion

This paper has proposed an efficient clinical support system called IAGA to discern the highly discriminative clinical features from the CTG dataset through ELM classifier to assess the fetal well-being. The classification results indicate that IAGA method has performed better in terms of classification accuracy and reduced feature count when compared with the previous works in the literature. The classification results are presented in terms of various performance measures like Sensitivity, Specificity, AUC, Overall Accuracy, *F*-measure, *G*-mean, and Kappa statistic. In order to demonstrate the effectiveness of this algorithm, five other benchmark datasets have been tested with the proposed IAGA search method, and the classification results are elaborated in detail. Also, these results are compared with other existing feature selection and feature reduction methods to potentiate its robustness. Observing the classification results obtained, it can be concluded that this decision support system has achieved the optimal solution obtained so far and has been instrumental for the obstetricians in predicting the fetal well-being more accurately.

## Figures and Tables

**Table 1:** List of features in CTG dataset.

S. number	Name of the features	Description
1	LB	FHR baseline (beats per minute)
2	AC	Number of accelerations per second
3	FM	Number of fetal movements per second
4	UC	Number of uterine contractions per second
5	DL	Number of light decelerations per second
6	DS	Number of severe decelerations per second
7	DP	Number of prolonged decelerations per second
8	ASTV	Percentage of time with abnormal short term variability
9	MSTV	Mean value of short term variability
10	ALTV	Percentage of time with abnormal long term variability
11	MLTV	Mean value of long term variability
12	Width	Width of FHR histogram
13	Min	Minimum of FHR histogram
14	Max	Maximum of FHR histogram
15	Nmax	Number of histogram peaks
16	Nzeros	Number of histogram zeros
17	Mode	Histogram mode
18	Mean	Histogram mean
19	Median	Histogram median
20	Variance	Histogram variance
21	Tendency	Histogram tendency: −1 = left asymmetric; 0 = symmetric; 1 = right asymmetric

**Table 2:** Specifications of IAGA.

Parameters	Specifications
Probability of crossover, *P*_*c*_	0.9
Type of crossover	Uniform crossover
Probability of mutation, *P*_*m*_	0.03
Type of mutation	Flip-bit mutation
Selection method	Stochastic selection
Number of runs	30
Length of chromosome	22
Population size	21
Number of elites	1
Maximum probability of crossover	0.9
Minimum probability of crossover	0.6
Maximum probability of mutation	0.1
Minimum probability of mutation	0.001

**Table 3:** Classification accuracies of all FS/FR methods using CTG dataset.

FS methods	Number of selected attributes	Accuracy obtained using ELM
PCA	16	92.14
	6	89.60
SFS	11	92.10
	6	91.44
SBS	11	92.71
	6	90.55
Basic GA	14	97.87
IAGA-M1	6	**93.61**
IAGA-M2	13	93.61

**Table 4:** Performance measures of CTG dataset.

Metrics	ELM	
	Acc. in %	*G*-mean
PCA	92.14	80.97
SFS	92.10	83.30
SBS	92.71	83.40
Basic GA	97.87	95.07
IAGA-M1	**93.61**	**85.57**
IAGA-M2	93.61	85.88

**Table 5:** Classification accuracies of classifiers using CTG dataset.

Classifier	Classification accuracy		
	With original features	After FS	% of increase
ELM	91.03	93.61	2.64

**Table 6:** Confusion matrix of CTG dataset.

Method	ELM		
	Pathological	Suspect	Normal
IAGA-M1	1620	61	10
	33	224	14
	2	10	152

**Table 7:** Comparison with previous works of all the datasets.

S. number	[Reference Number]	Features and methods	Selected features	Classifier	Accuracy
Multiclass classification					

CTG dataset					
1	[7]	ANFIS	—	—	97.15
2	[6]	GA	13	SVM	99.23
3	[8]	LS-SVM-PSO-BDT	—	SVM	91.62
4	Proposed study	IAGA-M1	6	ELM	**93.61 ± 0.42**

ES dataset					
1	[20]	IFSFS	21	SVM	98.61
2	[21]	Two-stage GFSBFS	20, 16, 19	SVM	100, 100, 97.06
3	[22]	GA based FS algorithm	16	BN	99.20
4	Proposed study	IAGA-M2	14	BN	**98.83 ± 0.12**

BT dataset					
1	[23]	Normalization	—	SVM	71.69
2	[24]	Electrical impedance spectroscopy	8		92
3	[25]	ACO and fuzzy system	—	SVM	71.69
4	Proposed study	IAGA-M2	3	ELM	**93.58 ± 0.42**

Binary Classification					

MEEI dataset					
1	[26]	30 acoustic features and PCA	17	SVM	98.1
2	[27]	LDA based filter bank energies	Not reported	LDA	85
3	[28]	22 acoustic features and IFS	16	SVM	91.55
4	Proposed study	22 acoustic features and IAGA	8	SVM	**100**

PD dataset					
1	[29]	GA	10	SVM	99
2	[30]	GA	9	*k*-NN	98.20
3	Proposed study	IAGA-M1	8	*k*-NN	**99.38 ± 0.22**

CAD dataset					
1	[31]	GA	9	SVM	83
2	[32]	WEKA filtering method	7	MLP	86
3	Proposed study	IAGA-M2	3	SVM	**83.23 ± 0.84**

**Table 8:** Overall comparison of fitness functions.

Datasets	Basic GA			IAGA-method 1			IAGA-method 2		
	Objv1	Objv2	Objv3	Objv1	Objv2	Objv3	Objv1	Objv2	Objv3
MEEI			*√*			*√*	×		
PD		×				*√*		×	
CAD	×					*√*			*√*
ES			*√*	×				×	
BT			*√*			*√*			*√*
CTG			*√*			*√*	×		
